# Nafion-stabilised bimetallic Pt–Cr nanoparticles as electrocatalysts for proton exchange membrane fuel cells (PEMFCs)†
†Electronic supplementary information (ESI) available. See DOI: 10.1039/c6ra16025e



**DOI:** 10.1039/c6ra16025e

**Published:** 2016-08-25

**Authors:** G. Gupta, S. Sharma, P. M. Mendes

**Affiliations:** a School of Chemical Engineering , University of Birmingham , Edgbaston , Birmingham , B15 2TT , UK . Email: p.m.mendes@bham.ac.uk

## Abstract

The current study investigated the unique combination of alloying (Pt with Cr) and Nafion stabilisation to reap the benefits of catalyst systems with enhanced catalytic activity and improved durability in PEMFCs. Pt–Cr alloy nanoparticles stabilised with Nafion were chosen in the current study owing to their higher stability in acidic and oxidising media at high temperatures compared to other Pt-transition metal alloys (*e.g.* Pt–Ni, Pt–Co). Two different precursor : reducing agent (1 : 10 and 1 : 20) ratios were used in order to prepare two different alloys, denoted as Pt–Cr 10 and Pt–Cr 20. The Pt–Cr 20 alloy system (with composition Pt_80_Cr_20_) demonstrated higher electrocatalytic activity for the oxygen reduction reaction compared to commercial Pt/C (TKK) catalysts. Accelerated stress tests and single cell tests revealed that Nafion stabilised alloy catalyst systems displayed significantly enhanced durability (only ∼20% loss of ECSA) compared with Pt/C (50% loss of ECSA) due to improved catalyst–ionomer interaction. Furthermore, the Pt–Cr 20 alloy system demonstrated a current density comparable to that of Pt/C making them promising potential electrocatalysts for proton exchange membrane fuel cells.

## Abstract 2

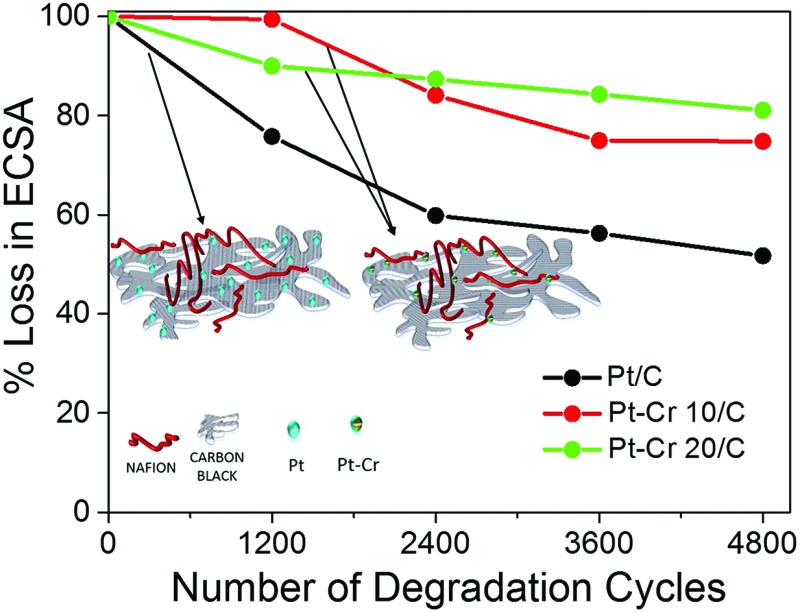
The current study investigated the unique combination of alloying (Pt with Cr) and Nafion stabilisation to reap the benefits of catalyst systems with enhanced catalytic activity and improved durability in PEMFCs.

## Introduction

In the last two to three decades, fuel cells have attracted a lot of attention in the search for alternative and green energy sources owing to their high efficiencies and low emissions. Proton exchange membrane fuel cells (PEMFCs) are promising candidates among the various next generation power sources for application in transportation, stationary and portable devices due to advantages like low operating temperature, quick start-up time, high current density and easy scale-up. However, large scale commercialisation of these systems is still hindered due to various problems related to expensive components, poor cathodic reaction kinetics and corrosion and/or aggregation of the catalyst.^1–3^ Pt-based electrocatalysts, commonly used in the PEMFC electrodes, are the main contributor to the high cost. High Pt loading, especially on the cathode side, to boost the sluggish oxygen reduction reaction (ORR)^1,2^ significantly adds to the system costs. The commercial catalyst is normally supported on high surface area carbon black. However, the porous structure of carbon black often makes many active catalytic sites inaccessible for the ORR leading to inefficient catalyst utilisation. Consequently, a significant amount of research focussing on decreasing the electrocatalyst loading while increasing the PEMFC efficiency has been carried out over the last decade by utilising various approaches, including (i) use of Pt group alloys to reduce Pt dependency, (ii) use of different carbon and non-carbon nanostructured supports to increase catalyst–support interaction, (iii) identifying the effect of particle shape and size on catalyst efficiency.^1–8^ However, more research is still required to achieve low cost, high-efficiency Pt catalyst systems for PEMFCs. Another approach, which has recently generated interest, is to improve the catalyst–ionomer interaction by impregnating proton-conducting polymers like Nafion into the electrocatalyst layer.^9–12^ This enhances the triple phase boundary between the ionomer, carbon support and the active catalytic sites resulting in improved ORR and hydrogen oxidation reaction (HOR) activity at the cathode and anode, respectively.

Various recent studies have reported the use of Pt and Pt based alloys dispersed in a polymer matrix for achieving enhanced catalytic properties.^9–12^ Liu *et al.* ^12^ synthesised Nafion stabilised Pt nanoparticles using alcohol reduction to realise higher oxygen reduction reaction activity compared to unsupported Pt black and commercial Pt/C catalyst. Sarma *et al.* ^9^ synthesised Nafion-stabilised PtRu/C catalysts for methanol oxidation reaction, which showed higher catalytic activity than a commercial E-TEK PtRu/C catalyst. Curnick *et al.* ^10,11^ prepared Nafion stabilised Pt nanoparticles using the borohydride reduction method and showed improved degradation behaviour as well as better utilisation when compared to the E-TEK catalyst. However, there have been limited studies investigating Pt–Cr alloys and their possible use as fuel cell catalysts. The main advantage offered by Pt–Cr alloy is its stablility in acidic and oxidizing media at high temperature. Moreover, Cr sits at the top of the volcano plot along with other transition metals (Ni, Co, Cu) suggesting significant potential to provide superior ORR activity.^13–15^ Yang *et al.* ^14^ studied the ORR kinetics of Pt–Cr alloy catalysts supported on Vulcan XC-72 and reported a significantly enhanced specific activity (a factor of ∼1.5–3) when compared to that of Pt/C. Antolini *et al.* ^15^ studied the electrocatalytic activities of different alloys of Pt–Cr for ORR in 1 M H_2_SO_4_ and 1 M H_2_SO_4_/1–3 M CH_3_OH solution using rotating disc electrode and reported improved activity as compared to Pt/C.

Herein, we report a novel approach, which combines the benefits of Nafion stabilisation with the useful effects of Pt–Cr alloy formation for application in PEMFC cathode. Nafion stabilised Pt–Cr alloy nanoparticles systems with two different alloying ratios were synthesised using wet chemical synthesis by borohydride reduction method. This unprecedented combination is expected to allow better catalyst–ionomer interaction, enable higher catalyst utilisation and enhance electrocatalytic activity and durability. We have based our studies on two different types of Nafion-stabilised Pt–Cr alloy nanoparticles in order to establish whether Nafion stabilised Pt–Cr alloy nanoparticles could provide superior catalytic activity and durability and if these properties could be dependent on the Pt : Cr ratio and Nafion content.

## Experimental

### Preparation of Nafion stabilised Pt–Cr nanoparticles

H_2_PtCl_6_ and CrCl_3_, as Pt and Cr precursors, respectively, were dissolved in ethanol in the molar ratio of 1 : 1 by continuous stirring at 500 rpm for 10 min. 10 wt% Nafion solution was added to the above mixture to attain a metal : Nafion ratio of 1 : 30. This mixture was stirred for another 30 min at 500 rpm to allow complete dispersion of Nafion in the ethanol solution. A freshly prepared solution of NaBH_4_ was then added to the above mixture which resulted in an instant colour change (from green to black). This confirmed that the metal precursors have been reduced. The product thus obtained was centrifuged (Sigma 3K 30, 4 °C, 2000 rpm) using acetone as the precipitation solvent to remove the excess Nafion. Following this, the product was re-dispersed using ultrasonication in a mixture of 1 : 4 v/v solution of acetone : water before centrifuging again. This process was repeated 3 times. The above procedure was used for the preparation of two alloys with different metal : reducing agent ratios (1 : 10 and 1 : 20) to allow variable degrees of reduction of the metal precursors. The alloys thus formed were labelled as Pt–Cr 10 and Pt–Cr 20, respectively. In the present work, the key parameter governing the alloying ratio is variation of the metal : reducing agent ratio. It is noteworthy that alloying ratio may also be controlled by varying Pt : Cr precursor ratio, though it was kept constant in the current study. The synthesis was repeated 4 times and samples from different batches were characterized using the below methods to confirm the repeatability of the process. Commercial Pt/C from TKK was used for comparison studies in fuel cell testing experiments.

### Material characterisation

The alloys formed were characterised using thermogravimetric analysis (TGA), transmission electron microscopy coupled with energy dispersive spectroscopy (TEM-EDX), X-ray diffraction (XRD) and X-ray photoelectron spectroscopy (XPS) to study the metal and Nafion content, particle size and distribution of the nanoparticles, the composition of the alloys and oxidation states of the various elements present.

### Transmission electron microscopy

The nanoparticles were deposited on Cu grids with carbon film on one side and examined using an FEI Tecnai TF20 coupled with Oxford Instruments INCA 350 energy dispersive X-ray spectroscopy (EDX) system operated at an accelerating voltage of 200 kV. Bright field images were taken at low and high resolution to analyse the size, size distribution and the structure of the nanoparticles. EDX was performed on the individual nanoparticles as well as over different areas to study the composition of the particles. The particle size was determined by analysing the high resolution images using ImageJ.

### X-ray diffraction (XRD)

Laboratory-source XRD (INEL XRD Equinox 3000 diffractometer) fitted with Cu Kα source, operating at 40 kV and 40 mA, was used to characterise the structure of the metal nanoparticles. The Pt–Cr nanoparticles were dried on a As-doped Si substrate for XRD measurement. The diffraction pattern was analysed using Match software. Instrumental broadening was determined using Si standard samples and particle size was estimated using the observed line broadening and Scherer equation at different planes of reflection.

### X-ray photoelectron spectroscopy (XPS)

XPS spectra were obtained on the AXIS Nova (Kratos Analytical) instrument based at University of Newcastle (NEXUS), UK. XPS experiments were carried out using a monochromatic Al Kα X-ray source (1486.7 eV) at a take-off angle of 90 degree to the surface plane. High-resolution scans of Pt 4f, C 1s, O 1s and Cr 2p were recorded using pass energy of 20 eV at a step size of 0.1 eV. Fitting of XPS peaks was performed using CASA XPS processing software. Sensitivity factors used in this study were: C 1s 1.00; O 1s 2.8; Pt 4f 7/2 8.65; Pt 4f 5/2 6.8; Cr 2p 3/2 7.69; Cr 2p 1/2 3.98.

### Thermogravimetric analysis (TGA)

Netzsch TG was used to study the amount of Nafion and metal (Pt/Cr) present in the samples. The samples were heated from room temperature to 800 °C at a heating rate of 10 °C min^–1^. The temperature of 800 °C was chosen to make sure the residual mass consists of Pt/Cr only while Nafion is completely burnt off so that no other impurities are present in the residue. TGA was also done for pure Nafion in order to compare it with the as synthesised alloy systems.

### Catalyst-ink preparation

The catalyst ink was prepared by supporting Pt–Cr nanoparticles on Vulcan XC-72 in the ratio of 1 : 1 in order to achieve the composition of 50 wt% Pt–Cr on Vulcan carbon in all cases. It was prepared in a solution of water and isopropanol (3 : 1). The ink was sonicated for 30 min to allow the mixing and homogenization of the catalyst nanoparticles with the carbon support.

### *Ex situ* electrochemical testing

The working electrode was prepared using a glassy carbon electrode (GCE) that was polished using alumina slurry successively with decreasing particle sizes of 1, 0.3 and 0.05 microns. 10 μl of the catalyst ink was then drop cast on the polished surface and completely dried in a vacuum oven at 40 °C for 12 hours. All the electrochemical testing was done at 25 °C in a 3 electrode cell, using an Autolab PGSTAT302N potentiostat (Metrohm) and a Pine rotating disc electrode (RDE) setup. The electrolyte in all cases was 0.1 M HClO_4_ solution prepared from 70% perchloric acid (Sigma-Aldrich). All the potentials were recorded with respect to normal hydrogen electrode (NHE) and Pt mesh was used as a counter electrode in all *ex situ* electrochemical measurements. Metal loadings of 20 μg cm^–2^ were used for all the CV and ORR experiments. All the tests were repeated at least 3 times to confirm the repeatability and homogeneity of the catalyst ink.

### Cyclic voltammetry (CV)

The HClO_4_ solution was purged with N_2_ gas for 20 min in order to saturate the solution with an inert gas before performing any measurements. CV scans were performed at 250 mV s^–1^ in a potential window from 0.05 V to 1.1 V to remove any contaminants that may be present on the surface of the catalyst. Steady state cyclic voltammograms were then recorded at a scan rate of 25 mV s^–1^ in the same potential range. Electrochemical surface area (ECSA) (m^2^ per g_Pt_) was then measured by integrating the hydrogen underpotential deposition (Hupd) region (0.05–0.4 V) from a CV obtained at scan rate of 25 mV s^–1^ using eqn (1).1
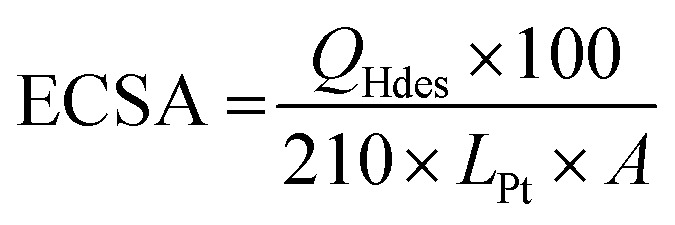
where *Q*_Hdes_ (μC) is the charge measured upon desorption of a monolayer of underpotentially-deposited (UPD) hydrogen, *L*_Pt_ is the Pt loading (μg cm^–2^), *A* is the geometric area of the GCE (cm^2^) and the value of 210 μC cm^–2^ corresponds to the charge passed during Hupd on a bulk polycrystalline Pt surface.

### RDE measurements of ORR activity

After the CV scans, the polarization curves were recorded between 0.05 to 1.2 V (*vs.* RHE) at a scan rate of 25 mV s^–1^ at room temperature. The sweeps were recorded at rotating speeds of 400, 800, 1200, 1600 and 2000 rpm. The solution was then purged with pure oxygen (O_2_) for 30 min to ensure that the solution was saturated and the same sweeps were recorded in O_2_.

### Accelerated stress testing

Accelerated stress testing (AST) was used to study the degradation behaviour of the standard catalyst as well as the Pt–Cr/C catalyst. The procedure involved recording the ECSA by cyclic voltammetry at 25 mV s^–1^ using the Hupd method before and after the potential cycling. Cycling consisted of square wave potential cycle from +0.6 V to 1.0 V holding for 1 s at each potential (S4†). These potentials were chosen in order to reproduce the open circuit and peak power output condition in the PEMFCs. The square wave potential step was chosen over linear potential sweep as this produced highest degradation loss due to Pt dissolution which shortens the test duration.^16^ This is also the most widely used test for the degradation study. The ECSA was measured after every 1200 cycles up to 4800 cycles. The 4800 cycles were selected for these *ex situ* studies since it provides^17–24^ information on the durability of the catalyst and whether there is the potential for the catalyst to be further developed.

### PEMFC single-cell testing

The single cell fuel cell testing was done using a Paxitech FCT-50S PEMFC test stand from Biologic. Membrane Electrode Assembly (MEA) consisted of a cathode gas diffusion layer, cathode catalyst, membrane, anode catalyst and anode gas diffusion layer. MEAs of size 4 cm × 4 cm were used for all the materials with a Nafion membrane of 5 cm × 5 cm. For the preparation of anodes, 1 wt% Nafion dispersion in iso-propyl alcohol (IPA) was painted on the catalyst side of commercial gas diffusion electrodes (Johnson Matthey, 0.4 mg_Pt_ per cm^2^ on SGL 34BC) followed by drying in a vacuum oven at 40 °C. Cathodes were prepared by hand painting the as-prepared Pt–Cr/C or commercial Pt/C (TKK) catalyst on the gas diffusion layer (Freudenberg C2) and drying under a UV lamp. The electrodes were left for drying in vacuum oven overnight at 40 °C and the catalyst loading was determined by measuring the weight change in the electrodes before painting and after painting and drying. Nafion 212 was used as the membranes between anode and cathode. The prepared anode and cathode GDLs and Nafion 212 membrane was then hot pressed (120 °C, 180 kPa, 120 s) to make the MEAs. The MEA was placed between two gaskets on either side and compressed between the graphite flow field plates with single cell serpentine channel geometry. Gold plated copper plates were used as current collectors and aluminium end plates were used to control the heating of the anode and cathode.

## Results and discussion

**Fig. 1:**
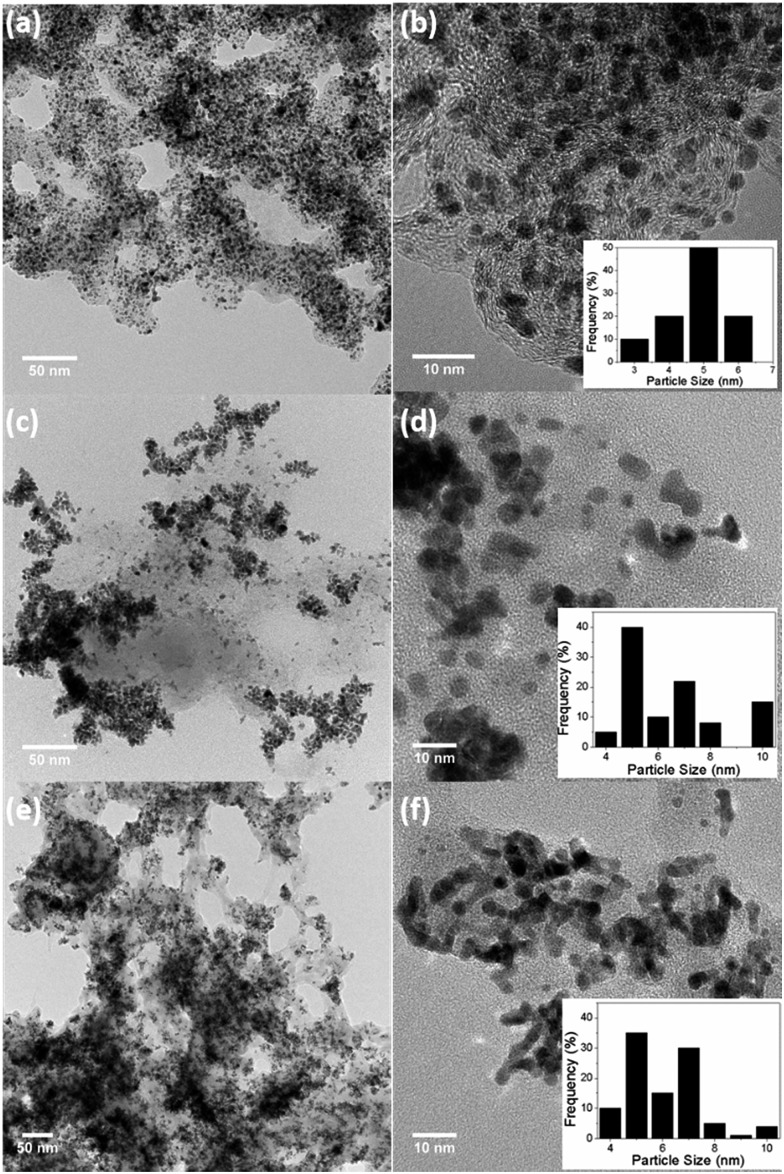
Bright field TEM images of (a, b) commercial Pt/C, (c, d) Pt–Cr 10 and (e, f) Pt–Cr 20 with particle size distribution shown in insets.

Pt–Cr alloy nanoparticles stabilised with Nafion were synthesised using borohydride reduction method with different precursor : reducing agent (1 : 10 and 1 : 20) ratios and were denoted as Pt–Cr 10 and Pt–Cr 20. Fig. 1 shows the bright-field TEM images of the commercial Pt/C (a, b), Pt–Cr 10 (c, d) and Pt–Cr 20 (e, f) samples. Nanoparticles with variable size distribution (as shown in the insets) from 4 to 10 nm were observed with a large fraction of particles around 6 ± 2 nm, indicating good size control can be achieved using the borohydride reduction process. A common feature observed in the images for both Pt–Cr samples, was the agglomeration of nanoparticles. Such agglomeration of nanoparticles has been reported in literature for Nafion stabilised samples^9,10,25^ as Nafion acts as a binder. Commercial Pt/C sample showed a uniform distribution of nanoparticles with average particle size of 4 ± 1 nm. Energy dispersive X-ray spectroscopy (EDX) was carried out on all the samples using area as well as particle scan for compositional analysis. The EDX graphs (Fig. S1†) revealed presence of 30 and 20 at% Cr in Pt–Cr 10 and Pt–Cr 20, respectively. A higher amount of Cr in Pt–Cr 10 than in Pt–Cr 20 samples was not completely unexpected as this is attributed to the relatively faster reduction of Cr precursor compared to Pt precursor leading to more Cr reduction by the time solid solution/alloying of Pt–Cr takes place. Similar observations have been reported for PtNiCr and PtRu alloys.^26,27^

**Fig. 2:**
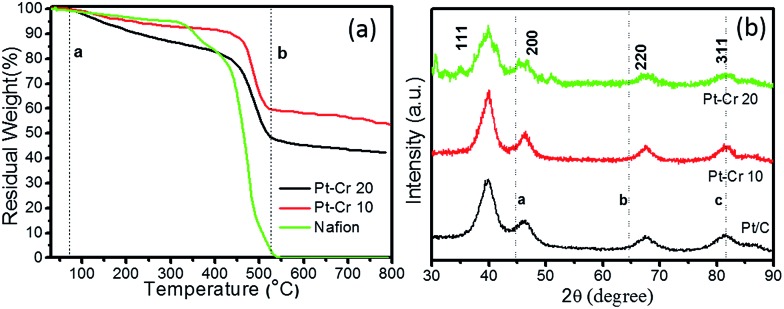
(a) TGA graph of Pt–Cr 10, Pt–Cr 20 and pure Nafion, (b) XRD graph of Pt/C, Pt–Cr 10 and Pt–Cr 20 (lines a, b and c are the peak positions for Cr).

Fig. 2a shows the TGA graphs for the two Pt alloys and pure Nafion. The decomposition of pure Nafion was found to occur between 75–550 °C. A small weight loss was observed in the temperature range of 75–300 °C which corresponds to the loss of residual water in the Nafion. A second more prominent weight loss was observed between 300 and 420 °C, which corresponds to the loss of sulphur dioxide from the Nafion.

The final curve in the temperature range 420–550 °C showed a drastic mass loss which corresponds to the complete decomposition of the Nafion polymer chain. These observations were in agreement with previous literature reports.^28,29^ Both Pt–Cr samples revealed a similar TGA profile showing the decomposition of Nafion from 75–500 °C with a steep change in mass after 450 °C. The amount of Nafion present in the samples was calculated using the weight loss between the two dotted lines (a and b) shown in the Fig. 2a. These calculations revealed that there is more Nafion in Pt–Cr 20 than in Pt–Cr 10. The residual weight at 800 °C was used to determine the amount of metal present in the samples. The metal loading was found to be 55 wt% and 43 wt% for Pt–Cr 10 and Pt–Cr 20, respectively. The TGA studies were repeated a minimum of three times for all samples and the error in the residual weight was found to be ±1 wt%.

Fig. 2b shows the X-ray diffraction graphs obtained for the three different catalysts. All samples revealed peaks at 2*θ* values of 39.8, 46.2, 67.6 and 81.5 which correspond to Pt (111), (200), (220) and (311), respectively. The dotted lines in the figures correspond to the peaks for Cr *i.e. a*(110), *b*(200), and *c*(211). Pt–Cr 20 showed more broadening of the Pt peaks compared to Pt–Cr 10 samples suggesting alloying of Pt and Cr. In most reported literature, alloying is identified either using peak shift and/or peak broadening as observed in the XRD analysis.^26,27,30,31^ As reported for Pt–Ru system by Hyun *et al.* ^27^ with the increase in the content of Ru, the Pt peak height decreases and broadening of the peaks occurs due to alloying. They also reported that degree of alloying increased with the increase in amount of reducing agent. Jeon *et al.* ^26^ reported peak broadening and height reduction for Pt in PtNiCr system with the increase in the concentration of reducing agent. The peak broadening was also attributed to the formation of alloys of PtNiCr. In the present study, the particle size is similar for the all the catalysts (ranging between 4–10 nm), thus the broadening of the peaks for Pt–Cr catalyst can be attributed to the formation of alloys. The lattice parameters for all samples as calculated from the XRD and TEM studies are compiled in Table 1. A small difference in the lattice parameter values can be due to different instruments and the accuracy of the measurement. The particle size calculated from the curves using the Scherer equation which takes into account the full width at half maximum (FWHM) for these catalysts is also shown in Table 1. The particle size varied from 4–10 nm for all the catalysts and is in agreement with the particle size calculated from the TEM analysis.

**Table 1:** parameters calculated from XRD and TEM analysis

Sample	Average particle size (TEM) nm	Particle size (XRD) nm	Lattice parameters (TEM) Å	Lattice parameters (XRD) Å
Pt/C	4 ± 1	6	3.92	3.92
Pt–Cr 10	6 ± 2	9	3.89	3.90
Pt–Cr 20	6 ± 2	8	3.85	3.89

XPS analysis was also carried out for the two Pt–Cr samples in order to identify the Pt : Cr ratio and oxidation states of the two metals. The survey spectrum for Pt–Cr 10 and Pt–Cr 20 (Fig. S2†) revealed peaks corresponding to Pt and Cr along with C, F, and O (due to the presence of Nafion). Minor impurity (1–5 at%) of Na was also identified in both samples (Table S1†). This however, is not expected to interfere with the performance of the alloy catalyst. The oxidation states of Pt and Cr elements were identified by peak fitting of the high-resolution elemental spectrum to obtain the component peaks. The ratio of Pt : Cr were also calculated for both the samples and were in agreement with the TEM-EDX results *i.e.* 30 at% Cr and 20 at% Cr in Pt–Cr 10 and Pt–Cr 20, respectively (Table S1†).

**Fig. 3:**
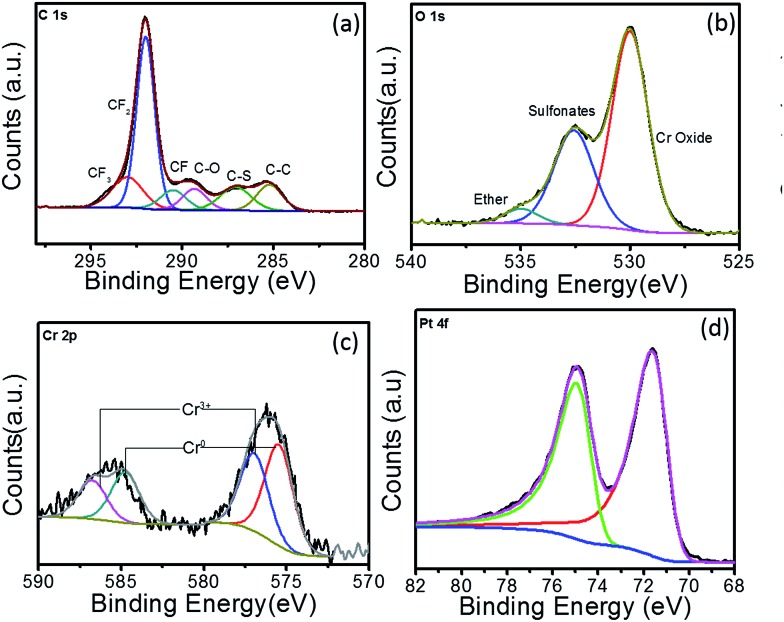
XPS spectra for Pt–Cr 10 showing high resolution elemental spectra of (a) C 1s, (b) O 1s, (c) Cr 2p and (d) Pt 4f.

Fig. 3 shows the high-resolution XPS spectra of C 1s, O 1s, Cr 2p and Pt 4f for Pt–Cr 10 samples along with the respective fitted peak components. All the peaks were normalised by shifting of the C–C peak to 285 eV. The different components in the C 1s spectrum (Fig. 3a) compare well with previous literature reports^32,33^ investigating the structure of the Nafion, which contains the CF_2_, CF_3_, CF, C–O and C–S bonds. Comparing the C 1s spectra of Pt–Cr 10 and pure Nafion (Fig. S3†) it can be seen that the component at 285 eV is absent in pure Nafion. The occurrence of C 1s component around 285 eV is attributed to the presence of adventitious carbon impurities during the synthesis procedure. However, there is a change in the ratio of CF components especially noticed in the decrease of CF_2_ component, which forms the backbone of Nafion structure. This suggests possible disintegration of the Nafion. The O 1s spectrum (Fig. 3b) of Pt–Cr 10 shows the presence of three different components at 529.9 eV, 532.6 eV and 535.1 eV, which correspond to the presence of metal oxide, sulfonate and ether groups, respectively.^32–34^ The presence of metal oxide can be confidently attributed to oxides of Cr which is further verified by the presence of Cr^3+^ components in Cr spectra (Fig. 3c).^35^ Note that no detection of Cr(vi) was observed that could potentially have a negative environmental impact. The partial disintegration of Nafion is confirmed from the change in the peak intensity of the sulfonate and ether groups. The peak intensity of the sulfonate group has increased significantly compared to pure Nafion and this can be due to the breakage of side chains of Nafion causing increase in the concentration of the sulfonate groups. Fig. 3c reveals the presence of two different components which were identified as Cr^0^ and Cr^3+^.^35,36^ Based on the deconvoluted components, it was determined that almost 40% of the Cr was oxidised during the synthesis. The oxidation of metal was unavoidable as the synthesis was carried out in ambient conditions without the use of any protective environment. However, this oxidation of Cr prevents the oxidation of Pt in this system. Fig. 3d shows the Pt 4f spectrum. The lower binding energy doublet with Pt 4f_7/2_ 71.2 eV agrees well with the published values of Pt metal.^35,37–42^ Further deconvolution of the spectrum confirmed presence of only one component pair corresponding to pure Pt and confirmed that Pt was not present in any other oxidation state. As suggested earlier, the absence of Pt oxides is attributed to the presence of Cr in the system, which has the tendency to oxidise more quickly, thus preventing the oxidation of Pt and in turn facilitating enhanced catalytic activity.

**Fig. 4:**
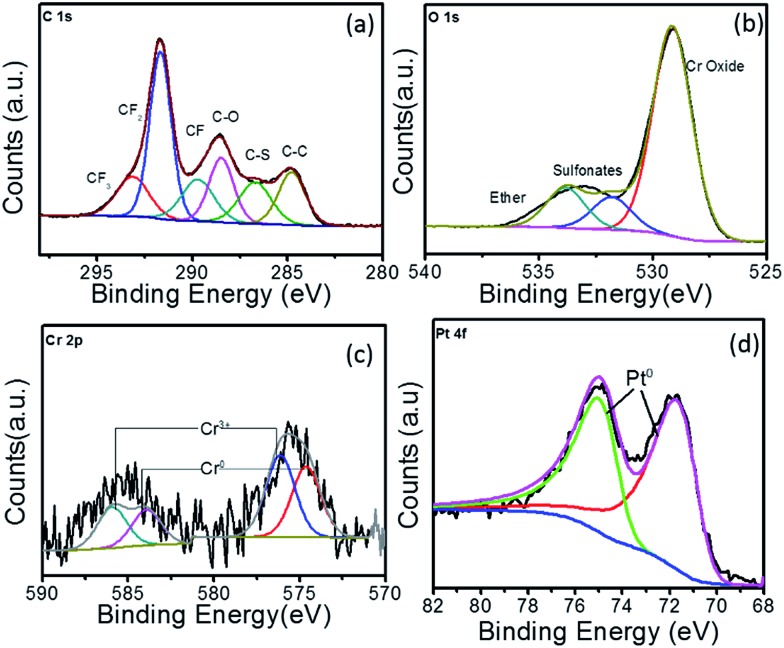
XPS spectra for Pt–Cr 20 showing high resolution elemental spectra of (a) C 1s, (b) O 1s, (c) Cr 2p and (d) Pt 4f.

The C 1s, O 1s, Cr 2p and Pt 4f high resolution XPS spectra for Pt–Cr 20 is shown in Fig. 4. The C 1s spectrum for this sample also showed the same components (*i.e.* CF_2_, CF_3_, CF, C–O and C–S bonds) as seen for Pt–Cr 10 (Fig. 3a), which confirmed the presence of Nafion. As mentioned earlier, C–C bond is present due to the adventitious carbon impurities during the synthesis. The concentration of CF groups is again changed and the intensity of CF_2_ peak has decreased suggesting some disintegration of Nafion. The O 1s spectrum (Fig. 4b) shows an increased intensity of the ether component, suggesting the breakage of side chains of Nafion containing ether group along with sulfonate groups in Pt–Cr 20. The O 1s spectra also revealed a metal oxide component peak (Fig. 4c) similar to the Pt–Cr 10 sample. Interestingly, the oxide content in this case appears to have increased to 54% as compared to 43% in Pt–Cr 10 even though the amount of Cr was less in Pt Cr 20. This could be due to the higher metal : reducing agent ratio used in this sample preparation which could lead to increase in Cr oxide formation. Similar to Pt–Cr 10, no oxidation of Pt was observed from the Pt 4f spectra for Pt–Cr 20.

Based on the various characterisation techniques (TGA, TEM, XRD and XPS) discussed above, it was estimated that the two systems synthesised in the current study *i.e.* Pt–Cr 10 and Pt–Cr 20 have 55 wt% and 43 wt% of metal, respectively. The amount of Cr present in the two systems, as estimated from XPS and EDX data is 30 at% and 20 at% for Pt–Cr 10 and Pt–Cr 20, respectively. The calculation for the amount of Pt and Cr present in both samples is given in the ESI.† This data was used as a baseline for carrying out the electrochemical experiments, which are discussed further in this study.

**Fig. 5:**
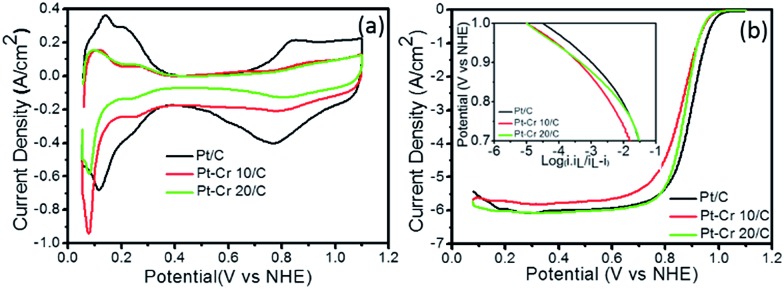
(a) Cyclic voltammetry and (b) linear sweep voltammetry (inset: Tafel plot) at 1600 rpm for Pt/C, Pt–Cr 10/C and Pt–Cr 20/C in 0.1 M HClO_4_ solution at 25 °C. The current density is normalised to Pt loadings estimated form the TGA, EDX and XPS analysis.

Fig. 5a shows the cyclic voltammograms of Pt/C, Pt–Cr 10/C and Pt–Cr 20/C in N_2_ saturated 0.1 M HClO_4_ solution at 25 °C. The values for the electrochemical surface area (ECSA) were calculated using eqn (1), which takes into consideration the area under the hydrogen desorption peak. The ECSA, specific and mass activity and Tafel slope values obtained for all the catalysts are reported in the Table 2. A lower ECSA value was observed for Pt–Cr samples as compared to Pt/C which can be attributed to loss of area due to agglomeration and presence of larger particles in the Pt–Cr samples. The lower ECSA for Pt–Cr 20 compared to Pt–Cr 10 may be ascribed to the presence of higher amount of Nafion in the sample, which can shield the metal nanoparticles.

**Table 2:** Parameters calculated from electrochemical characterisation

	Pt/C	Pt–Cr 10/C	Pt–Cr 20/C
ECSA (m^2^ per g_Pt_)	61	27	19
Tafel slope (mV per decade)	75	73	62
Specific activity at 0.9 V (mA per cm_Pt_^2^)	0.45	0.42	0.69
Mass activity at 0.9 V (mA per mg_Pt_)	260	118	130

Fig. 5b shows the linear sweep voltammograms of the three catalyst–support systems (Pt/C, Pt–Cr 10/C and Pt–Cr 20/C) recorded at a rotation speed of 1600 rpm. The linear sweep voltammetry graph was used to calculate the mass normalised Tafel plots for both the Pt–Cr catalyst systems in comparison with the commercial Pt/C catalyst system (inset Fig. 5b). Specific and mass activity for all the three catalyst systems was calculated at 0.9 V. Pt–Cr 20/C alloy showed higher specific activity in comparison to Pt/C catalyst. However, there was no improvement in mass activity. The specific activity enhancement can be attributed to two factors, (1) improved triple phase boundaries due to Nafion stabilisation and (2) formation of Pt–Cr alloys.^10,12^ The Nafion stabilization of the Pt–Cr nanoparticles occurs by the combination of steric and electrostatic mechanisms. When carbon support is added, it results in a very effective distribution of Pt–Cr nanoparticles that have good contact with the proton conducting network *i.e.* improved triple phase boundary. There is also the possibility that Nafion swelling^43–45^ can contribute to the fast diffusion of protons, thus resulting in enhanced nanoparticle performance.

**Fig. 6:**
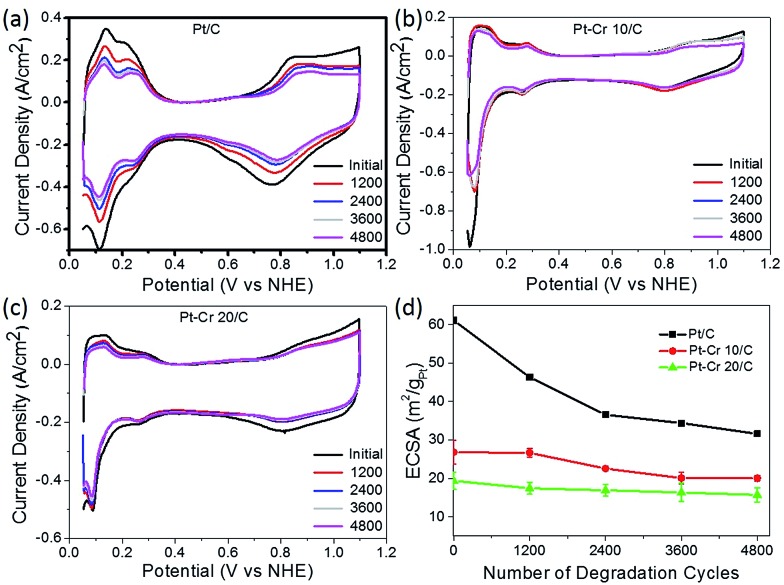
Cyclic voltammograms with different cycles for (a) Pt/C, (b) Pt–Cr 10/C and (c) Pt–Cr 20/C and (d) comparison of loss of electrochemical surface area for all the samples with potential cycling. All samples were tested a minimum of 3 times and standard deviation is shown in (d).

All three catalyst systems were subjected to degradation studies using cyclic voltammetry. CVs were recorded after every 1200 cycles to calculate the amount of degradation for each catalyst (Fig. 6a–c), which in turn provided the reduction in the ECSA. The ECSA values obtained for each catalyst over the 4800 cycles are plotted in Fig. 6d. A significant loss of performance was observed for the commercial Pt/C catalyst after 1200 (25%) and 2400 (40%) compared to only around 15% and 13% after 2400 cycles for Pt–Cr 10 and Pt–Cr 20, respectively. The degradation rate of Pt–Cr 10 is almost negligible during the initial 1200 cycles and becomes constant only after 3600 cycles. The degradation trend for Pt–Cr 10 and Pt–Cr 20 is different, and this can be due to the different amount of Nafion in both set of nanoparticles. The degradation rate of Pt/C slowed down after 2400 cycles, reducing 10% more after 4800 cycles. Although the ECSA of Pt/C is still higher than Pt–Cr alloys after 4800 cycles, Pt/C has already lost 50% of its initial active area. Pt–Cr alloys have retained 75–80% of the initial active area, which shows the better durability of these catalysts. The degradation of Pt/C is known to occur due to various reasons, including (1) increase in the Pt particle size, (2) dissolution of the catalysts and (3) carbon corrosion.^46–50^ On the other hand, the Nafion stabilized Pt–Cr catalyst systems showed only a 20–25% loss of ECSA. This enhanced durability is attributed to (1) the presence of Pt–Cr alloys and (2) Nafion stabilization of the catalyst nanoparticles. As evident from the TEM images, the Pt–Cr nanoparticles are in close coordination due to Nafion stabilization and this can help inhibit the Pt dissolution, diffusion and sintering processes resulting in better durability of these catalysts. It has also been reported in literature that Nafion stabilization helps in preventing catalyst degradation.^9,10,12,51,52^ Limited literature on Pt–Cr alloy^5,14,15,53–56^ systems, in the absence of Nafion stabilisation, reported ECSA values which were similar to the initial ECSA values observed for Pt–Cr systems in this study. However, these reports did not investigate the durability of Pt–Cr alloys either by studying the change in ECSA or by any other method.

**Fig. 7:**
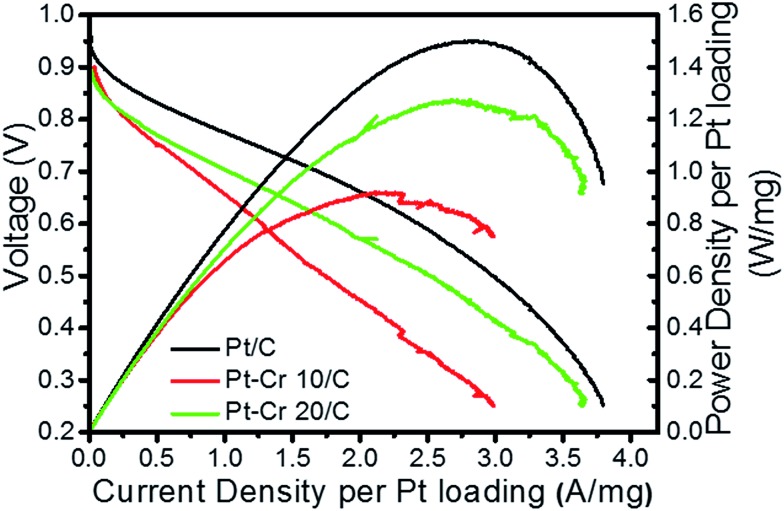
Current density and power density curves for Pt/C, Pt–Cr 10/C and Pt–Cr 20/C in single cell fuel cell testing condition after preparation of membrane electrode assembly. The curves are normalised by Pt loading.

Fig. 7 shows the relationship between voltage and power density with respect to current density for the three catalyst systems. The current and power density are normalised to the Pt loading (0.3–0.4 mg cm^–2^). A figure without Pt normalisation is shown in the ESI (S5†). Pt–Cr 20/C shows a comparable performance as compared to the Pt/C catalyst and the slight difference can be due to the following reasons: (i) the amount of Pt in alloy was not sufficient to allow uniform distribution of Pt on the MEA resulting in some areas with low or no catalyst concentration; (ii) the agglomeration of nanoparticles, which also led to reduced ECSA (as seen from the RDE testing); (iii) the amount of Nafion that was used in these systems is higher than the optimum value required for such a system and may block some of the catalytic sites. As mentioned earlier, Nafion stabilization results in improved contact between Pt nanoparticles and carbon support, resulting in the formation of an extended triple phase boundary. Thus, the better performance of the Pt–Cr 20 as compared to Pt–Cr 10 is likely a result of the higher amount of Nafion in the former that leads to a more effective triple phase boundary.

Theoretically, the trend of *ex situ* results (*i.e.* ECSA and specific activity) should follow the trend of *in situ* results (*i.e.* power density). However, this is not always the case due to several factors that can vary between *ex situ* and *in situ* techniques,^57,58^ including operation temperature (*ex situ* studies are carried out at room temperature while *in situ* tests are carried out at 80 °C), distribution of the nanoparticles on the MEAs and thickness of the catalyst layer. In the present case, we reported the agglomeration of nanoparticles, which can be more visible in the *ex situ* results due to the very small amount of catalyst used. The *in situ* results are expected to better reflect upon the behaviour of the catalyst as the general *in situ* set-up is much closer to the operation of a real fuel cell.

The better performance of Pt–Cr 20/C catalyst as compared to Pt–Cr 10/C can be attributed to the more convenient Pt availability for the reaction. Moreover, the presence of higher amount of Cr_2_O_3_ in Pt–Cr 20/C can further enhance the activity of the alloy catalyst. The presence of metal oxides is considered to improve the performance of the catalyst by the dissolution of metal oxide, thereby, allowing exposure of Pt for participation in electrochemical reactions.^2,59,60^ From the *ex situ* and *in situ* testing of the catalysts it can be said that Pt–Cr 20/C shows a better catalytic activity and durability in comparison to the commercial Pt/C catalyst.

## Conclusions

Pt–Cr alloy nanoparticles stabilised in Nafion with average particle size of 6 ± 2 nm and Cr concentrations of 20–30 at% were successfully synthesised.

The *ex situ* electrochemical tests revealed that the specific activity of Pt–Cr 20/C is almost twice that of Pt/C. The degradation studies carried out *ex situ* using accelerated stress testing also confirmed that the Nafion stabilised Pt–Cr alloys have superior durability with only 20–25% loss in ECSA after 4800 cycles as compared to commercial Pt/C which lost almost 50% of its active area during the same time. The superior performance is ascribed to improved alloy–catalyst–ionomer interaction leading to minimal catalyst dissolution (one of the primary reasons for catalyst loss in fuel cells). The *in situ* testing of the catalysts showed that the performance of Pt–Cr 20/C in terms of current density is comparable to that of the commercial Pt/C making them promising potential catalysts for PEMFCs.

The performance of these alloys can be further enhanced by optimisation of the amount of Nafion in the system. Thus, with the use of this system, it is possible to achieve reduced Pt loading and enhanced performance and durability for the PEMFC system while improving its economic viability. Future studies will look into optimisation of Nafion content to further enhance current density while maintaining the durability of the system.

## References

[cit1] Handbook of Fuel Cells: Fundamentals, Technology, Applications, ed. W. Vielstich, A. Lamm and H. A. Gasteiger, Wiley, New York, 2003.

[cit2] PEM Fuel Cell Electrocatalysts and Catalyst Layers: Fundamentals and Applications, ed. J. Zhang, Springer, 2008.

[cit3] Wang Y., Chen K. S., Mishler J., Cho S. C., Adroher X. C. (2011). Appl. Energy.

[cit4] Sharma S., Pollet B. G. (2012). J. Power Sources.

[cit5] Antolini E. (2003). Mater. Chem. Phys..

[cit6] Bing Y., Liu H., Zhang L., Ghosh D., Zhang J. (2010). Chem. Soc. Rev..

[cit7] Markovic N. M., Schmidt T. J., Stamenkovic V., Ross P. N. (2001). Fuel Cells.

[cit8] Yu W., Porosoff M. D., Chen J. G. (2012). Chem. Rev..

[cit9] Sarma L. S., Lin T. D., Tsai Y.-W., Chen J. M., Hwang B. J. (2005). J. Power Sources.

[cit10] Curnick O. J., Mendes P. M., Pollet B. G. (2010). Electrochem. Commun..

[cit11] Curnick O. J., Pollet B. G., Mendes P. M. (2012). RSC Adv..

[cit12] Liu Z., Tian Z. Q., Jiang S. P. (2006). Electrochim. Acta.

[cit13] Mukerjee S., Srinivasan S. (1993). J. Electroanal. Chem..

[cit14] Yang H., Alonso-Vante N., Leger J.-M., Lamy C. (2004). J. Phys. Chem. B.

[cit15] Antolini E., Salgado J. R. C., Santos L. G. R. A., Garcia G., Ticianelli E. A., Pastor E., Gonzalez E. R. (2006). J. Appl. Electrochem..

[cit16] Uchimura M., Kocha S. S. (2007). ECS Trans..

[cit17] Liu Y., Xu C. (2013). ChemSusChem.

[cit18] Jayasayee K., Veen J. A. R. V., Manivasagam T. G., Celebi S., Hensen E. J. M., de Bruijn F. A. (2012). Appl. Catal., B.

[cit19] Ignaszak A., Teo C., Ye S., Gyenge E. d. (2010). J. Phys. Chem. C.

[cit20] Lim B., Lu X., Jiang M., Camargo P. H., Cho E. C., Lee E. P., Xia Y. (2008). Nano Lett..

[cit21] Chen Z., Waje M., Li W., Yan Y. (2007). Angew. Chem..

[cit22] Han B., Xu C. (2014). Int. J. Hydrogen Energy.

[cit23] Duan H., Hao Q., Xu C. (2015). J. Power Sources.

[cit24] Savych I., Subianto S., Nabil Y., Cavaliere S., Jones D., Roziere J. (2015). Phys. Chem. Chem. Phys..

[cit25] Curnick O., Doctor of Philosophy, University of Birmingham, UK, 2012.

[cit26] Jeon M. K., Zhang Y., McGinn P. J. (2009). Electrochim. Acta.

[cit27] Hyun M.-S., Kim S.-K., Lee B., Peck D., Shul Y., Jung D. (2008). Catal. Today.

[cit28] Baturina O. A., Aubuchon S. R., Wynne K. J. (2006). Chem. Mater..

[cit29] Samms S. R., Wasmus S., Savinell R. F. (1996). J. Electrochem. Soc..

[cit30] Cullity B. D., Elements of X-ray diffraction, Addison-Wesley Publishing Company, Inc., Reading, Massachusetts, 1956.

[cit31] Suryanarayana C. and Norton M. G., X-Ray Diffraction: A Practical Approach, Plenum Press, New York, 1998.

[cit32] Schulze M., Lorenz M., Wagner N., Gülzow E. (1999). Fresenius. J. Anal. Chem..

[cit33] Martínez de Yuso M. V., Neves L. A., Coelhoso I. M., Crespo J. G., Benavente J., Rodríguez-Castellón E. (2012). Fuel Cells.

[cit34] Tan S., Belanger D. (2005). J. Phys. Chem. B.

[cit35] Gupta G., Iqbal P., Yin F., Liu J., Palmer R. E., Sharma S., Leung K. C.-F., Mendes P. M. (2015). Langmuir.

[cit36] Salvi A. M., Castle J. E., Watts J. F., Desimoni E. (1995). Appl. Surf. Sci..

[cit37] Moulder J. F., Stickle W. F., Sobol P. E. and Bomben K. D., Handbook of X-ray Photoelectron Spectroscopy, Physical Electronics, Inc., Minnesota, United States of America, 1995.

[cit38] Sharma S., Ganguly A., Papakonstantinou P., Miao X., Li M., Hutchison J. L., Delichatsios M., Ukleja S. (2010). J. Phys. Chem. C.

[cit39] Aricò A. S., Shukla A. K., Kim H., Park S., Min M., Antonucci V. (2001). Appl. Surf. Sci..

[cit40] Casella I. G., Desimoni E. (1996). Electroanalysis.

[cit41] Shukla A. K., Neergat M., Bera P., Jayaram V., Hegde M. S. (2001). J. Electroanal. Chem..

[cit42] Sarkar A., Manthiram A. (2010). J. Phys. Chem. C.

[cit43] Choi P., Jalani N. H., Datta R. (2005). J. Electrochem. Soc..

[cit44] Bauer F., Denneler S., Willert-Porada M. (2005). J. Polym. Sci., Part B: Polym. Phys..

[cit45] Satterfield M. B., Majsztrik P. W., Ota H., Benziger J. B., Bocarsly A. B. (2006). J. Polym. Sci., Part B: Polym. Phys..

[cit46] Yu X., Ye S. (2007). J. Power Sources.

[cit47] Stevens D. A., Dahn J. R. (2005). Carbon.

[cit48] Shao Y., Yin G., Gao Y. (2007). J. Power Sources.

[cit49] Shao Y., Yin G., Gao Y., Shi P. (2006). J. Electrochem. Soc..

[cit50] Wang Z.-B., Zuo P.-J., Chu Y.-Y., Shao Y.-Y., Yin G.-P. (2009). Int. J. Hydrogen Energy.

[cit51] Cheng N., Mu S., Pan M., Edwards P. P. (2009). Electrochem. Commun..

[cit52] Yin S., Mu S., Lv H., Cheng N., Pan M., Fu Z. (2010). Appl. Catal., B.

[cit53] Yang H., Alonso-Vante N., Lamy C., Akins D. L. (2005). J. Electrochem. Soc..

[cit54] Yano H., Kataoka M., Yamashita H., Uchida H., Watanabe M. (2007). Langmuir.

[cit55] Mukerjee S., Srinivasan S., Soriaga M. P., McBreen J. (1995). J. Phys. Chem..

[cit56] Min M.-k., Cho J., Cho K., Kim H. (2000). Electrochim. Acta.

[cit57] Kongkanand A., Subramanian N. P., Yu Y., Liu Z., Igarashi H., Muller D. A. (2016). ACS Catal..

[cit58] Huang Y., Zhang J., Liu Y., Subramanian N. P., Wagner F. T., Jorne J. and Li J., 2011, 1009 1020, 10.1149/1.3635633.

[cit59] Seo A., Lee J., Han K., Kim H. (2006). Electrochim. Acta.

[cit60] Park K.-W., Choi J.-H., Kwon B.-K., Lee S.-A., Sung Y.-E., Ha H.-Y., Hong S.-A., Kim H., Wieckowski A. (2002). J. Phys. Chem. B.

