# Supplementary material for: Nafion-stabilised bimetallic Pt–Cr nanoparticles as electrocatalysts for proton exchange membrane fuel cells (PEMFCs)
Source: RSC Adv. 2016 Aug 25;6(86):82635–43. doi: 10.1039/c6ra16025e (PMC5059791; doi:10.1039/c6ra16025e)
Supplement: Supplementary file 1 [file RA-006-C6RA16025E-s001.pdf]

Electronic Supplementary Material (ESI) for RSC Advances.
This journal is © The Royal Society of Chemistry 2016

Supporting Information

Nafion-stabilised bimetallic Pt-Cr nanoparticles as electrocatalysts for proton exchange membrane fuel cells (PEMFCs)

G. Gupta[a], S. Sharma[a] and P.M. Mendes[a,*]

[a]*School of Chemical Engineering, University of Birmingham, Edgbaston, Birmingham, B15 2TT, UK.*

Correspondence Address

Professor Paula M Mendes

School of Chemical Engineering

University of Birmingham

Edgbaston, Birmingham, B15 2TT (UK)

Tel: Int. code +(121) 414-5343

Email: p.m.mendes@bham.ac.uk

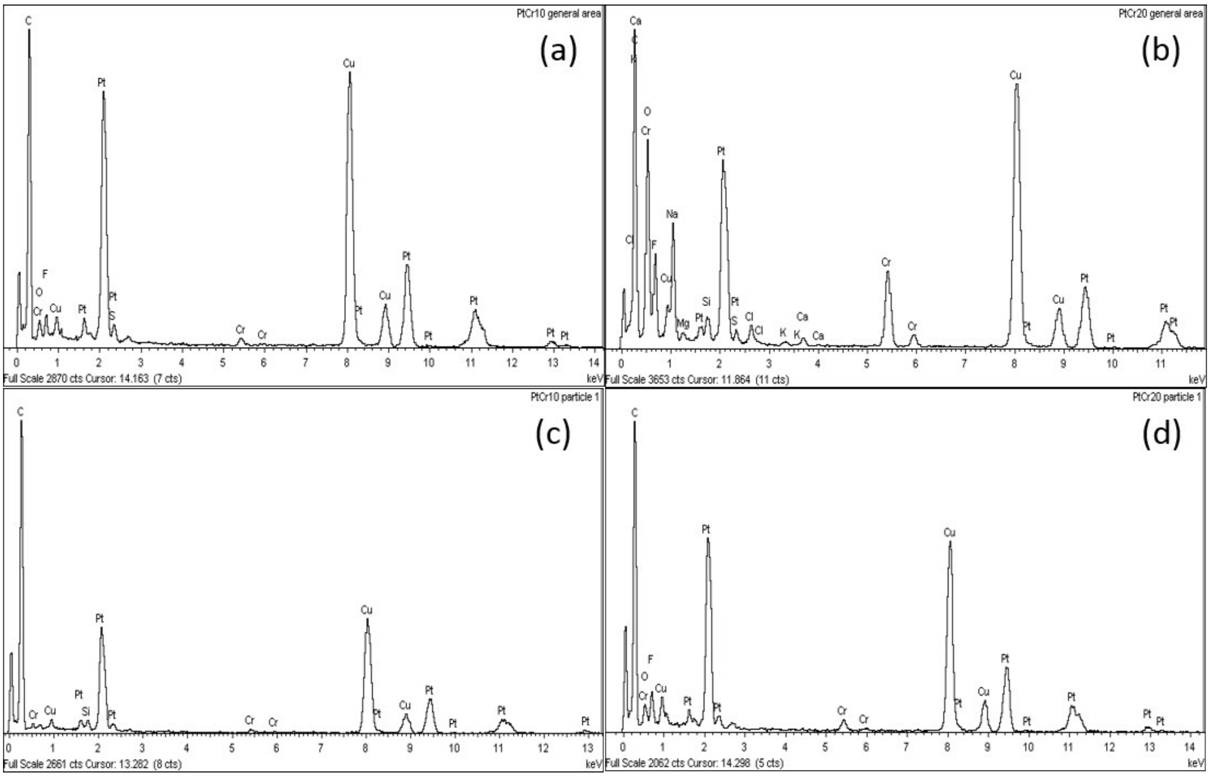

Figure S1: EDX graphs of Pt-Cr 10 (a- area, c-particle) and Pt-Cr 20 (b- area, d- particle) samples.

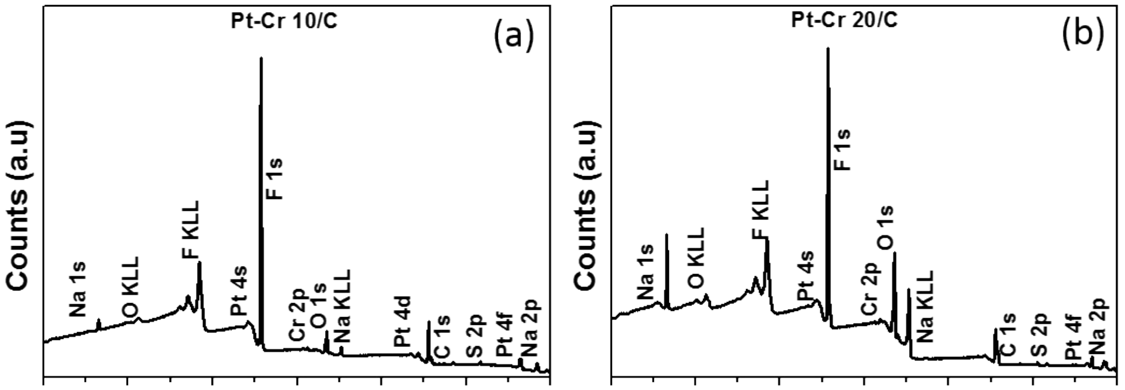

Figure S2: Survey spectra of Pt-Cr 10 and Pt-Cr 20 samples.

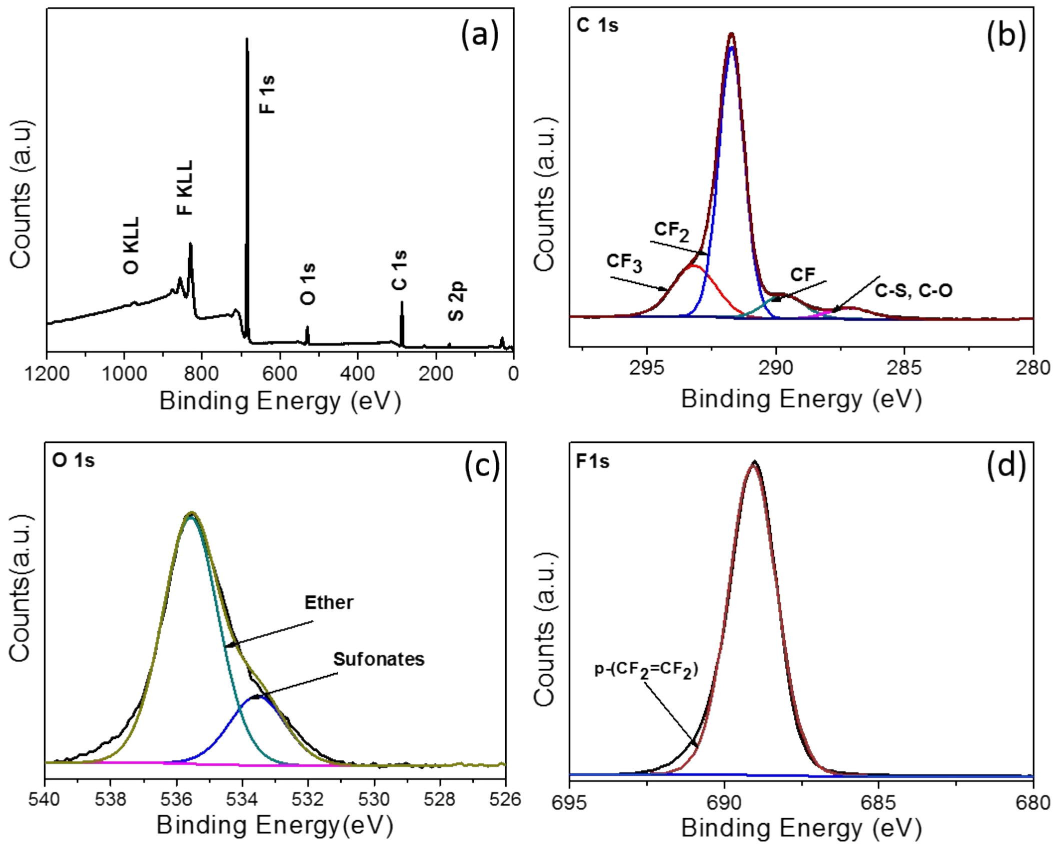

Figure S3: XPS spectra of Nafion.

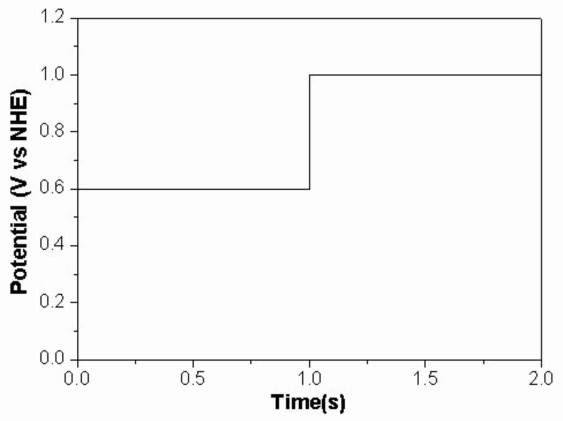

Figure S4: Accelerated stress testing profile.

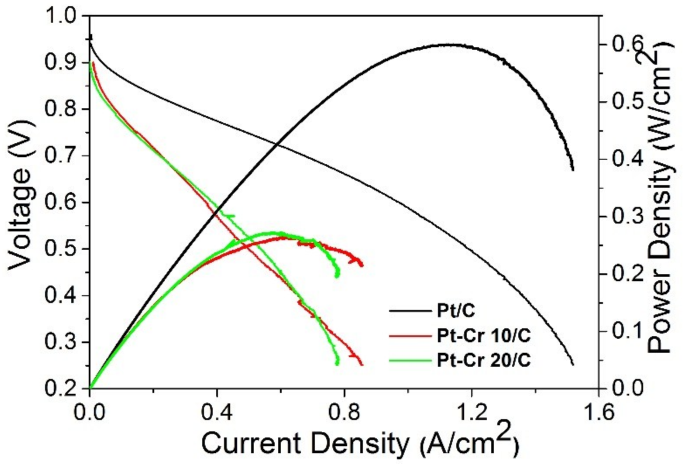

Figure S5: Current density and power density curves for Pt/C, Pt-Cr 10/C and Pt-Cr 20/C in single cell fuel cell testing condition after preparation of membrane electrode assembly.

Table S1: (a) Atomic ratio of elements present in the samples as calculated from XPS, (b) ratio of Pt and Cr present in the samples.

**Sample / Elements**	**Pt-Cr 10**	**Pt-Cr 20**
**Pt (at. %)**	0.63 ± 0.007	0.64 ± 0.004
**Cr (at. %)**	0.27 ± 0.005	0.16 ± 0.003
**C (at. %)**	35.02 ± 0.35	33.03 ± 0.51
**O (at. %)**	9.01 ± 0.25	20.01 ± 0.43
**F (at. %)**	52.97 ± 0.42	39.96 ± 0.56
**Na (at. %)**	1.05 ± 0.04	5.36 ± 0.06
**S (at. %)**	1.05 ± 0.03	0.84 ± 0.04

Sample	Ratio of Pt:Cr
Pt-Cr 10	70:30
Pt-Cr-20	80:20

Sample calculation for estimating the amount of Pt and Cr in the samples:

1) **Pt-Cr 10**

Assuming amount of sample = 5 mg

From TGA, amount of metal= 55 wt.%

Amount of metal = $\frac{55}{100} * 5$ = 2.75 mg

From XPS & EDX, Amount of Pt = 70 at.% = 89.75 wt.%

Amount of Cr = 30 at.% = 10.25 wt.%

Amount of Pt in the sample = $\frac{89.75}{100} * 2.75$ = 2.47 mg

Amount of Cr in the sample = $\frac{10.25}{100} * 2.75$ = 0.28 mg

2) **Pt-Cr 20**

Assuming amount of sample = 5 mg

From TGA, amount of metal= 43 wt.%

Amount of metal = $\frac{43}{100} * 5$ = 2.15 mg

From XPS & EDX, Amount of Pt = 80 at.% = 93.75 wt.%

Amount of Cr = 20 at.% = 6.25 wt.%

Amount of Pt in the sample = $\frac{93.75}{100} * 2.15$ = 2.02 mg

Amount of Cr in the sample = $\frac{6.25}{100} * 2.15$ = 0.13 mg
